# Local perforator island flaps in post-traumatic reconstruction of middle third of the leg

**DOI:** 10.1007/s11751-014-0189-8

**Published:** 2014-03-09

**Authors:** Jerry R. John, Ramesh Kumar Sharma

**Affiliations:** Department of Plastic Surgery, Post graduate Institute of Medical Education and Research, Chandigarh, 160012 India

**Keywords:** Trauma, Open fracture, Tibia, Perforator island flap, Lower extremity, Tissue defect

## Abstract

Perforator flaps have been introduced for coverage of local and distant defects. Various designs of this flap are possible, but their role in the setting of trauma is debated. We report that it is possible to raise these flaps in cases of post-traumatic lower limb reconstruction with good results. Consideration must be given to the type of movement that is planned vis-a-vis the number of perforators identified.

## Introduction

Gustilo has classified compound fractures of the leg depending on the extent of skin and soft tissue loss, the nature of injury, extent of bone exposure and vascular status of the leg [1]. The grade IIIb open fracture of the tibia typically has exposed bone which is devoid of periosteal or endosteal supply. It requires flap cover to prevent desiccation or sepsis and for the fracture to heal. Reconstructive surgeons divide the fractured leg into the upper third, middle third and lower third, depending on the area where the tibia is exposed. Although no universally applicable algorithm for soft-tissue reconstruction exists, there are a few commonly practiced flaps for each of these territories [2]. The gastrocnemius flap is widely preferred for a defect in the upper third. Similarly, soleus or hemisoleus flaps have been used for coverage of defects in the middle third. Proximally or distally based fasciocutaneous flaps are also common options for upper- and middle-third defects. In contrast, microsurgical free flaps are the first choice for defects in the lower third due to the paucity of undamaged local tissue [2].

Salmon, Manchot and Taylor have pioneered flaps based on increasingly small but reliable blood vessels—perforators [3, 4]. The origin of perforator flaps was an extension of the concept that the integument of the body can be divided into angiosomes [5]. A perforator flap can be defined as a flap supplied by perforator vessels that originate from a deep vascular system and reach the flap by coursing through either a muscle or an intermuscular septum [6].

Since the reports by Taylor et al., numerous anatomical and clinical studies have been published in support of different free and pedicled perforator flaps. However, local perforator-based island flaps used in a post-traumatic setting have not gained universal acceptance. Critics of this technique cite the presence of degloving and possible traumatic injury to local perforators as contraindications to performing flaps based on them.

We have performed perforator-based island flaps in a traumatic setting with considerable success. Two such instances are described below. The purpose of the article is to elucidate the merits and demerits of local perforator-based flap designs.

## Case 1

**Fig. 1:**
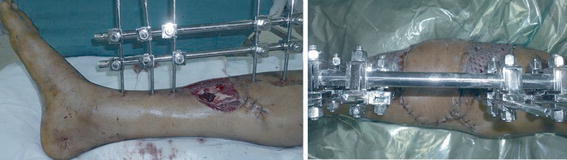
*Left* Exposed fracture site on the medial aspect of middle third of tibia. *Right* Postoperative view. The flap was islanded based on peroneal perforator and propelled medially

A young male presented with fracture of both bones over the middle third of leg. The post-debridement defect measured 5 × 4 cm in size. No local degloving was present. A perforator flap was raised from the lateral side. It was based on a single perforator from the peroneal vessels. The distal portion of the flap was rotated 90° to cover the defect. The secondary defect was skin grafted (Fig. 1).

## Case 2

**Fig. 2:**
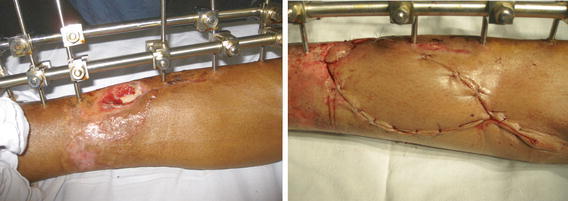
*Left* Exposed fracture site. *Right* V–Y advancement flap from the medial upper leg

A young male presented with a fracture of the middle third of the tibia. The wound measured 4 × 2 cm with the fracture site forming the floor of the wound. A perforator-based island flap was raised on branches from the posterior tibial vessels. The flap was advanced in a V-to-Y fashion into the defect (Fig. 2).

## Discussion

A local perforator-based pedicled flap permits a certain degree of flexibility in operative planning and decision-making. One only needs to identify at least one viable perforator in the vicinity of the defect. Preoperative Doppler-marking of the perforator allows planning of incisions and flap designs. However, it is almost impossible to quantify or limit the area or territory supplied by a particular perforator owing to several factors: the diameter of the perforating vessel may vary or a part of the flap can fail to sustain itself on blood supply from a relatively distant perforator. This may lead to partial necrosis of the perforator flap in the postoperative period. Such partial necrosis may be due to part of the flap being elevated from the zone of injury.

The design of local pedicled perforator-based flaps can be broadly described by the movement that the flap makes. A flap can have either an axial rotatory movement or a purely linear advancement. A perforator-based island flap which has rotated on its axis up to 180° can be described as a propeller flap [7]. Those flaps which do not have such a rotatory movement can be described as advancement flaps [8].

V–Y advancement perforator-based flaps were first described by Venkataramakrishnan et al. [9] for various leg defects post-malignancy. The flap replaces ‘like with like’ and leaves only a linear scar as the donor site defect. These flaps were recommended in selected cases of post-traumatic leg reconstruction by Niranjan et al. [10]. The size of the defect has to be small as the advancement derived is only a couple of centimetres. Two advancement flaps may be fashioned from either side to cover a larger defect over the anteromedial tibia. In the case of advancement flaps, the portion of the flap that finally covers the defect will be the tissue which was originally adjacent to the defect. In a post-traumatic setting, the viability of this tissue can be precarious. Elevation of this tissue as part of the advancement flap may lead to necrosis and leave the fracture site exposed even if the rest of the flap survives. The advantage of the propeller design is that the part of the flap which is recruited to cover the exposed fracture site is remote from the zone of trauma. The tissue adjacent to the defect is moved away from the defect, and it is of minor concern if this part necroses as the fracture would still be covered by the viable part of the flap.

Pedicled propeller flaps can cover larger defects as compared to advancement flaps. However, for a propeller design perforator-based flap, only one perforator can be kept intact. All the other perforators would have to be sacrificed to facilitate movement of the flap. The surgeon has to take particular care that the twist of the intact vascular pedicle is distributed over a length of at least 3 cm [11]. This is not the case with advancement designs in which a number of perforators can be preserved and the flap advanced without risk of the perforators twisting on their axis. The likelihood of survival of an advancement flap is greater as more perforators can be relied upon to nourish the flap.

These considerations are of vital importance in a trauma setting: (a) there is no substitute for thorough debridement of the injured area; (b) subdermal bleeding from the flap-edge lying adjacent to the defect has to be brisk; (c) the vascular pedicle supplying the flap has to be at least 1 mm in diameter and meticulously preserved; (d) tension on the flap-edge suture line has to be avoided and (e) an acute kink of the perforator has to be prevented while insetting the flap.

## Conclusion

Local pedicled perforator-based flaps can be reliably performed in a post-trauma setting. No particular flap design can be deemed superior as each has its own merits and risks. The operating surgeon should carefully consider the options of design when exploring for perforators.

## References

[1] Gustilo RB, Mendoza RM, Williams DN (1984). Problems in the management of type III (severe) open fractures: a new classification of type III open fractures. J Trauma.

[2] Kasabian AK, Karp NS, Thorne CH (2007). Lower-extremity reconstruction. Grabb and Smith’s plastic surgery.

[3] Manchot C (1983). The cutaneous arteries of the human body.

[4] Salmon M, Taylor GI, Razaboni RM (1994). Arterial anastomotic pathways of the extremities. Book 2.

[5] Taylor GI, Pan WR (1998). Angiosomes of the Leg: anatomic study and clinical implications. Plast Reconstr Surg.

[6] Blondeel PN, Van Landuyt KH, Monstrey SJ, Hamdi M, Matton GE, Allen RJ, Dupin C, Feller AM, Koshima I, Kostakoglu N, Wei FC (2003). The “Gent” consensus on perforator flap terminology: preliminary definitions. Plast Reconstr Surg.

[7] Pignatti M, Ogawa R, Hallock GG, Mateev M, Georgescu AV, Balakrishnan G, Ono S, Cubison TCS, D’Arpa S, Koshima I, Hyakusoku H (2011). The, “Tokyo” consensus on propeller flaps. Plast Reconstr Surg.

[8] Dancey A, Blondeel PN (2010). Technical tips for safe perforator vessel dissection applicable to all perforator flaps. Clin Plast Surg.

[9] Venkataramakrishnan V, Mohan D, Villafane O (1998). Perforator based V–Y advancement flaps in the leg. Br J Plast Surg.

[10] Niranjan NS, Price RD, Govilkar P (2000). Fascial feeder and perforator-based V–Y advancement flaps in the reconstruction of lower limb defects. Br J Plast Surg.

[11] Wong CH, Cui F, Tan BK, Liu Z, Lee HP, Lu C, Foo CL, Song C (2007). Nonlinear finite element simulations to elucidate the determinants of perforator patency in propeller flaps. Ann Plast Surg.

